# The Story of the Insane from Year to Year

**Published:** 1894-12-08

**Authors:** 


### THE STORY OF THE INSANE FROM YEAR TO YEAR.

(7th Series.)

### ISLE OF MAN ASYLUM.

There were 212 patients in this asylum at the close of the financial year, being an increase of one during the preceding twelve months. There were 41 admissions, 18 recoveries, and 19 deaths. The recoveries, calculated on the admissions, stand at the very creditable percentage of 43·9 and the deaths at 9·4. Hereditary tendency caused the insanity in 10 of the year's admissions, and we do not notice "drink" given as a cause, which we hope may be construed to mean that the Manx are a sober race. Cerebro-spinal diseases are responsible for 8 of the deaths and consumption for 1 only. Influenza made its appearance, and is responsible for 3 of the deaths. "The Board have much pleasure in stating that Dr. Richardson continues to manage the asylum in a most efficient and satisfactory manner." After certain deductions, the weekly cost of maintenance stands at 6s. 7d. per head.

### NEWCASTLE BOROUGH ASYLUM.

On December 31st, 1893, this asylum contained 449 patients, being one more than on January 1st. There were 119 admissions, 40 recoveries, and 60 deaths; and these numbers yield a percentage of 33·6 recoveries and 13·5 deaths. As regards the causes of insanity in the year's admissions, drink comes first with 33; domestic trouble drove 18 insane. Hereditary influence accounts for 27, and "previous attacks" for 16. Of the deaths 32 were from cerebral or spinal diseases, and nine from consumption. The asylum is full, if not more than full, and additions have been commenced for the accommodation of 360 patients. As the City of Newcastle is a rapidly increasing one, these additions are by no means excessive, and the numbers of patients will increase perhaps more quickly than the committee anticipate. The asylum would seem to be in excellent order, and the visitors say they "have much pleasure in recording their satisfaction with the management of the asylum by Dr. Callcott." The weekly rate of maintenance is a fraction ove 8s. per head.

### OXFORD ASYLUM AT LITTLEMORE.

The numbers went down from 529 to 517, but there were 13 absent on trial; 103 patients were admitted, 36 recovered, and 46 died. The recoveries stand at 34 per cent. on the admissions, and the deaths 9 per cent. on the average number resident. Hereditary tendency and intemperance in drink are respectively responsible for 15 and 14 of the admissions. Domestic trouble for 2 and 31 are said to be unknown; 14 of the deaths are put down to disease of the lungs. The female side of the asylum is quite full, and a male dormitory has to be occupied by female patients; and a dissolution of the partnership between Oxford and Windsor has been spoken of. We are pleased to note that Mr. Cobb, the clerk of the works, obtained a pension of two-thirds of his salary. The charge to the unions is 7s. 6d. per week per head.

